# Performance of wrist-worn home sleep apnea testing (watch-PAT) among individuals with chronic insomnia: a comparative study with polysomnography

**DOI:** 10.3389/fneur.2026.1817165

**Published:** 2026-06-10

**Authors:** Chuan Shi, Jinmei Luo, Rong Huang, Yi Xiao

**Affiliations:** Department of Pulmonary and Critical Care Medicine, Peking Union Medical College Hospital, Chinese Academy of Medical Science and Peking Union Medical College, Beijing, China

**Keywords:** diagnosis, home sleep apnea testing, insomnia, polysomnography, sleep apnea

## Abstract

**Background:**

While comorbid insomnia and sleep apnea are frequently encountered in clinical practice, the accuracy of home sleep apnea testing in this particular scenario has long been overlooked. Our study aims to evaluate the performance of a wrist-worn device in diagnosing sleep apnea among individuals with chronic insomnia.

**Methods:**

Participants with chronic insomnia suspected of having sleep apnea were consecutively enrolled at a sleep center. Single-night Watch-PAT 200 monitoring was conducted in parallel with attended in-lab polysomnography.

**Results:**

Recordings from 44 participants (41% female, age 49.1 ± 12.7 years) with a wide range of sleep duration (222.9 ~ 461.4 min) and severity of sleep apnea [apnea–hypopnea index (AHI) 0.3 ~ 83.9/h] were analyzed. Using AHI_PAT_ ≥ 15/h as the threshold, the sensitivity, specificity, and area under the receiver operating characteristic curve for identifying moderate-to-severe obstructive sleep apnea (OSA) were 77.3% (95% CI 54.2–91.3%), 100.0% (95% CI 81.5–100.0%), and 0.92 (95% CI 0.80–0.98), respectively. Diagnostic agreement defined by clinically oriented criteria was reached in 36 (82%) subjects. The Watch-PAT overestimated total sleep time and rapid eye movement sleep duration by 19.9 min (limits of agreement −48.1 ~ 87.9 min) and 37.2 min (limits of agreement −26.1 ~ 100.6 min), respectively.

**Conclusion:**

Among individuals with chronic insomnia, the wrist-worn home sleep apnea testing maintained acceptable diagnostic performance for identifying moderate-to-severe OSA. Sleep duration and staging parameters provided by the device should be interpreted with caution.

## Introduction

Obstructive sleep apnea (OSA) is a clinical condition characterized by repeated episodes of upper airway collapse during sleep, affecting approximately one-seventh of the world’s adult population (1). Without proper management, OSA may lead to increased risks of various cardiovascular and metabolic conditions, adverse brain health outcomes, and impaired work performance (2, 3). Beyond the impact on personal wellbeing, the socioeconomic burden of OSA is far-reaching, owing in large part to massive underdiagnosis (4). Therefore, timely screening and accurate diagnosis still play central roles in this field of medical practice.

While it has been well-recognized that chronic insomnia is highly prevalent among individuals with OSA (5), this co-occurrence has recently received increased attention due to worse outcomes relative to each condition alone (6, 7). Comorbid insomnia and sleep apnea (COMISA) has been associated with poorer daytime function, greater risks for medical and psychological morbidities, and increased overall mortality (8), representing a clinical challenge. To date, a series of studies have examined the effect of insomnia on the acceptance and adherence to OSA therapy (7). Less is known, however, about how insomnia affects the screening and diagnosis of OSA.

In-laboratory overnight polysomnography (PSG) is considered the optimal diagnostic study for patients with suspected OSA with comorbid sleep disorders. However, resources for this labor-intensive and space-consuming test are limited. In addition, sleeping in unfamiliar environments with complex devices often hinders patients with poor sleep. By contrast, home sleep apnea testing (HSAT) provides a more convenient and cost-efficient approach, but the lack of electroencephalography, electrooculography, and electromyography in most devices prevents accurate documentation of total sleep time (TST) and sleep stages. This technological limitation often leads to underestimation of the severity of OSA, which might be worsened in the presence of insomnia (9).

Watch-PAT is a wrist-worn HSAT system that measures respiratory events using peripheral arterial tone (PAT) and pulse signals rather than the classical approach of airflow and respiratory effort (10). The assessment of sleep/wake state, as well as sleep staging, is based on actigraphy and PAT patterns (11, 12). The diagnostic accuracy of Watch-PAT has been validated in sleep center settings (13), population cohorts (14), and various cardiovascular and respiratory conditions (15, 16). The utilization of the device has been standardized by the American Academy of Sleep Medicine Scoring Manual since version 2.2 (17). It would be promising to assess both respiratory events and sleep parameters with a device that minimizes sleep interference. However, the value of Watch-PAT for diagnosing OSA and evaluating sleep/wake state among individuals with chronic insomnia has not been thoroughly investigated. The current study aims to assess the performance of Watch-PAT in this population.

## Materials and methods

### Participants

The current study consecutively enrolled individuals with suspected OSA and underlying chronic insomnia referred to the sleep breathing disorder center of Peking Union Medical College Hospital (PUMCH), a university-affiliated tertiary teaching hospital in Beijing, China. The enrollment period was from July 2022 to July 2024. Chronic insomnia was defined according to the third edition of the International Classification of Sleep Disorders (ICSD-3).

Patients were excluded for any of the following reasons: (1) age <18 years or > 70 years; (2) received treatment for sleep apnea prior to enrollment; (3) medical histories (non-sinus arrhythmia, pacemaker implantation, peripheral vascular disease, peripheral neuropathy) or regular use of medications (alpha-receptor blockers, nitrates) that may affect peripheral arterial tone (PAT) signals; and (4) abnormalities of the hands and/or wrists resulting in the inability to wear the Watch-PAT.

The sample size required was estimated through the formula $n=\frac{{Z_{\frac{\alpha }{2}}}^{2}\times \mathrm{Se}\times (1-\mathrm{Se})}{d^{2}\times P}$ where *n* is the sample size, *Z* is the statistic corresponding to the confidence level of 1−α , *Se* is the pre-determined sensitivity, *d* is the maximum marginal error, and *P* is the expected prevalence of OSA among participants. We adopted previously reported sensitivity data for Watch-PAT in a Chinese cohort (18) and assumed a *P* of 80% based on our experience from our sleep center. The confidence level and marginal error were set at 95 and 10%, respectively. Allowing for device intolerance and/or technical failure in 20% of enrolled participants, the final sample size for enrollment was estimated to be 50.

All participants provided written consent. The study was approved by the Research Ethics Committee of Peking Union Medical College Hospital (Approval No. K22C1402) and performed in accordance with the Declaration of Helsinki.

Before performing the PSG and Watch-PAT, participants were asked to complete electronic forms that consisted of: (1) demographic information; (2) Insomnia Severity Index (ISI); and (3) Epworth Sleepiness Scale (ESS). The ISI is a validated and widely used 7-item questionnaire assessing the nature, severity, and impact of insomnia, with a total score ranging from 0 to 28 (19). The ESS measures the level of daytime sleepiness by assessing the likelihood of falling asleep in eight daily situations. Each item is rated from 0 (would never doze) to 3 (high chance of dozing), yielding a total score ranging from 0 to 24 (20). Assistance, if needed, was provided by a sleep physician during the survey.

### Polysomnography

All participants underwent full-night in-laboratory PSG (Embla N7000; Natus Medical Incorporated, Orlando, USA; or SOMNO HD; SOMNO Medics AG, Randersacker, Germany). Electroencephalogram, electrooculogram, chin electromyogram, electrocardiogram, airflow, respiratory effort, pulse oxygen saturation (SpO_2_), snoring, and body position were continuously recorded. Data were recorded using software that accompanies the device (RemLogic for Embla N7000; DOMINO for SOMNO HD) and scored by two experienced sleep technicians following the AASM Manual for the Scoring of Sleep and Associated Events (version 2.6).

Apnea was scored as a drop in airflow ≥ 90% from the pre-event baseline lasting at least 10 s. Hypopnea was defined as a drop in airflow ≥ 30% lasting at least 10 s, with associated ≥ 3% desaturation from baseline or arousal. The apnea–hypopnea index (AHI) was defined as the number of apnea and hypopnea events per hour. The oxygen desaturation index (ODI) was defined as the number of desaturations per hour. Total sleep time (TST), sleep latency, sleep efficiency, wake after sleep onset (WASO), rapid eye movement (REM) sleep duration, the percentage of each sleep stage, the lowest oxygen saturation by pulse oximetry (LSpO_2_), and the percentage of time spent with SpO_2_ below 90% (T90) were also collected.

### Watch-PAT

For each participant, the Watch-PAT 200 U (Itamar Medical Ltd., Caesarea, Israel) recording was completed in parallel with the PSG. The Watch-PAT 200 U is a wrist-worn device with an attached finger-mounted PAT and oximetry probe. An additional snore and body position sensor was placed under the sternal notch. Data from Watch-PAT were analyzed using the zzzPAT software (version 4.6.72.6). After automated analysis, manual scoring was performed according to a proposed algorithm that improves the accuracy of sleep staging and the identification of respiratory events (21). Different rules for retaining or deleting breathing events were applied during REM and non-REM sleep. To enable a parallel comparison, the recording time of the PSG was used to select the time range for Watch-PAT data output. In addition, the PSG and Watch-PAT scoring were performed in a blinded manner by independent scorers.

### Data analysis

Data were analyzed using MedCalc version 20.014 (MedCalc Software Ltd., Ostend, Belgium). The normality of the variables was tested using the Shapiro–Wilk test. Demographic, clinical, and sleep study data were summarized as means and standard deviations, medians and interquartile ranges, or exact counts and percentages. Comparisons between PSG-derived and Watch-PAT-derived parameters were performed with paired t-tests or Wilcoxon tests. Statistical agreement between the two methods was analyzed with the intra-class correlation coefficient (ICC) and Bland–Altman plots. ICC was calculated using a two-way mixed-effects model (absolute agreement, single measurement). Sleep latency and AHI were compared after logarithmic transformation to achieve a normal distribution. ICC values were interpreted as follows: <0.5 for poor agreement, 0.5–0.75 for moderate agreement, 0.75–0.9 for good agreement, and >0.90 for excellent agreement.

Using PSG data as the gold standard, the diagnostic performance of Watch-PAT was then evaluated by sensitivity, specificity, and receiver operating characteristic (ROC) curves at different AHI_PSG_ cutoffs. Mild, moderate-to-severe, and severe OSA were defined as AHI_PSG_ ≥ 5/h, ≥ 15/h, and ≥ 30/h, respectively. Because a bias of minimal clinical significance (e.g., AHI of 29/h vs. 31/h) may result in diagnostic misclassification in standard sensitivity and specificity assessments, diagnostic accuracy was also evaluated using clinically oriented criteria specifically developed for HSAT (22). In brief, *diagnostic agreement* was reached when both AHI_PSG_ and AHI_PAT_ were ≥ 40/h, or when the AHI bias was within 10/h when AHI_PSG_ < 40/h. *Overestimation* and *underestimation* of AHI were considered when the bias of AHI was greater than 10/h (unless both were ≥ 40/h). When Watch-PAT *underestimates* AHI and simultaneously overestimates TST, a sleep-time-adjusted AHI_PAT_ was calculated, based on the hypothesis that no respiratory events occur during the wake period misjudged as sleep. The sleep-time-adjusted AHI_PAT_ was then compared with AHI_PSG_ using the same criteria to determine whether the underestimation of AHI is primarily due to the overestimation of TST. All statistical tests were two-sided, and *p*-values < 0.05 were considered statistically significant.

## Results

### Clinical and demographic data

**Figure 1:**
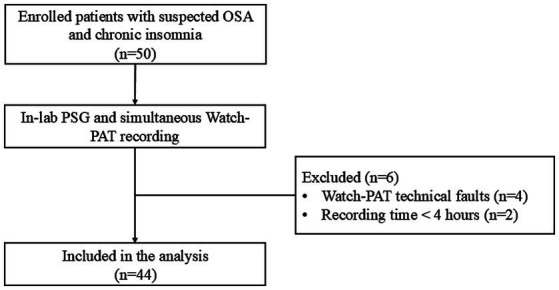
STROBE flowchart of the study. OSA, obstructive sleep apnea; PSG, polysomnography; STROBE, strengthening the reporting of observational studies in epidemiology.

A total of 50 individuals were recruited for simultaneous PSG and Watch-PAT testing. Data from six subjects were excluded due to technical faults of either method (*n* = 4), recording time of less than 4 h (*n* = 2) (Figure 1). To assess for potential selection bias, baseline clinical characteristics of the 6 excluded participants were compared with those of the 44 analyzed participants. There were no significant differences in mean age (49.1 vs. 43.2 years, *p* = 0.902), BMI (25.5 vs. 25.5 kg/m^2^, *p* = 0.999), or sex distribution. The demographic and clinical characteristics of the 44 included subjects are summarized in Table 1.

**Table 1:** Demographic and clinical characteristics of the study population.

Characteristics	Total (*N* = 44)	AHI_PSG_ < 15/h (*N* = 22)	AHI_PSG_ ≥ 15/h (*N* = 22)	*P*
Demographic characteristics				
Age (years), mean±SD	49.1 ± 12.7	48.9 ± 11.7	49.4 ± 13.8	0.888
Sex, *n* (%)				0.069
Female	18 (41)	12 (55)	6 (27)	
Male	26 (59)	10 (45)	16 (73)	
Marital status, *n* (%)^#^				0.346
Unmarried	5 (12)	2 (9)	3 (14)	
Married	36 (84)	17 (77)	19 (86)	
Divorced/separated/widowed	2 (5)	2 (9)	0 (0)	
Clinical characteristics				
Hypertension, *n* (%)	9 (21)	2 (9)	7 (32)	0.065
Diabetes mellitus, *n* (%)	5 (11)	1 (5)	4 (18)	0.159
Dyslipidemia, *n* (%)	10 (23)	5 (23)	5 (23)	1.000
Difficulty initiating sleep, *n* (%)	18 (41)	9 (41)	9 (41)	1.000
Difficulty maintaining sleep, *n* (%)	20 (45)	10 (46)	10 (46)	1.000
Early awakening, *n* (%)	27 (61)	14 (64)	13 (59)	0.760
Daily caffeine intake, *n* (%)	20 (46)	12 (55)	8 (36)	0.231
Regular use of sleep aids, *n* (%)	9 (21)	5 (23)	4 (18)	0.712
Current smoker, *n* (%)	3 (7)	1 (5)	2 (9)	0.554
Body mass index (kg/m^2^), median [IQR]	24.6 [22.7, 27.8]	23.6 [22.0, 25.9]	26.3 [23.7, 28.7]	0.016
Neck circumference (cm), mean±SD^#^	37.4 ± 4.3	35.9 ± 4.0	38.9 ± 4.1	0.018
Waist circumference (cm), mean±SD^#^	90.8 ± 12.6	85.5 ± 10.0	96.0 ± 12.9	0.005
Epworth Sleepiness Scale, mean±SD ^#^	11.5 ± 5.6	10.8 ± 5.0	12.2 ± 6.1	0.412
Insomnia Severity Index, mean±SD^#^	16.0 ± 4.5	16.6 ± 4.0	15.4 ± 5.0	0.399

# Available in 43 participants; SD, standard deviation; IQR, interquartile range.

The participants covered a wide range of PSG-derived sleep duration from 222.9 min to 461.4 min, with a mean of 370.0 min. Broad ranges were also present for sleep latency (0.2 ~ 120.5 min), efficiency (52.3 ~ 99.6%), and REM duration (0 ~ 113.5 min). The median AHI_PSG_ was 15.1 [6.2, 26.8] /h (range 0.3 ~ 83.9/h). The number of participants with AHI_PSG_ < 5/h, 5/h ≤ AHI_PSG_ < 15/h, 15/h ≤ AHI_PSG_ < 30/h, and AHI_PSG_ ≥ 30/h was 8 (18%), 14 (32%), 13 (30%), and 9 (20%), respectively.

### Statistical agreement

The statistical agreement was first assessed using ICC (Table 2). The ICC on log-transformed AHI values indicated good agreement between the two methods. There was moderate agreement for the TST, WASO, and the lowest SpO_2_ assessed by PSG and Watch-PAT. The agreement for REM sleep duration and log-transformed sleep latency was poor.

**Table 2:** Comparison and agreement of polysomnography and Watch-PAT-derived parameters.

Parameters	Polysomnography	Watch-PAT	Intraclass correlation coefficient (95% CI)
Total sleep time (min), mean±SD	370.0 ± 51.0	389.9 ± 46.5**	0.69 (0.41–0.84)
Sleep latency (min), median [IQR]	11.5 [5.5, 32.0]	15.3 [4.6, 34.9]	0.27 (−0.04–0.53) ^#^
Wake after sleep onset (min), mean±SD	64.3 ± 45.4	45.3 ± 40.3**	0.60 (0.31–0.78)
REM sleep duration (min), mean±SD	59.3 ± 22 0.8	97.1 ± 36.5**	0.25 (−0.09–0.54)
AHI (/h), median [IQR]	15.1 [6.2, 26.8]	11.7 [5.8, 20.3]*	0.80 (0.65–0.89) ^#^
Lowest SpO_2_ (%), mean±SD	86.3 ± 6.1	83.3 ± 6.0**	0.53 (0.22–0.73)

*p < 0.05; **p < 0.001; ^#^ calculated after log transformation; AHI, apnea–hypopnea index; CI, confidence interval; IQR, interquartile range; ODI, oxygen desaturation index; REM, rapid eye movement; SD, standard deviation.

**Figure 2:**
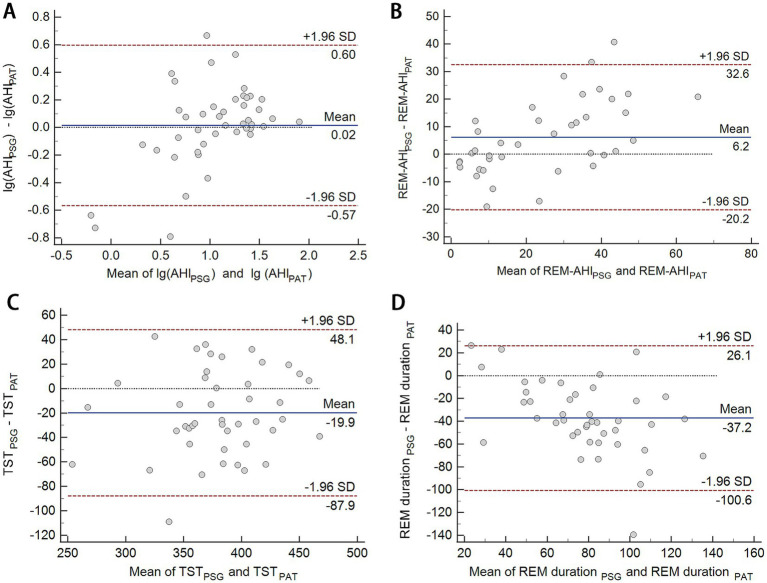
Bland–Altman plots of the apnea–hypopnea index and sleep duration measured using polysomnography and Watch-PAT. **(A)** Log-transformed apnea–hypopnea index, **(B)** rapid eye movement sleep-specific AHI, **(C)** total sleep time, and **(D)** rapid eye movement sleep duration. The *X*-axis represents the mean; the *Y*-axis represents the difference (polysomnography measured value minus Watch-PAT measured value). The blue line indicates the mean bias, while the red dashed lines indicate 95% limits of agreement. REM, rapid eye movement; SD, standard deviation; TST, total sleep time.

In the Bland–Altman analysis, the mean difference for AHI was 0.02 on the log scale (Figure 2A). After back-transformation, in 95% of cases, the AHI_PAT_/ AHI_PSG_ ratio would fall between 0.27 and 3.94. The mean biases of TST, REM sleep duration, and REM stage-specific AHIs (REM-AHIs) were 19.9 min (limits of agreement: −48.1 and 87.9 min), 37.2 min (limits of agreement: −26.1 and 100.6 min), and 6.2/h (limits of agreement: −32.6 and 20.2/h), respectively (Figures 2B–D).

### Diagnostic performance

**Figure 3:**
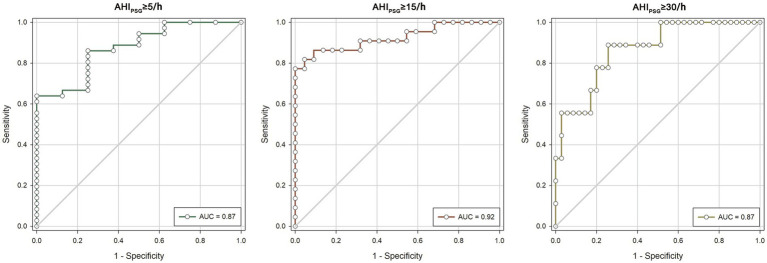
Receiver operating characteristic curves of Watch-PAT for diagnosis of obstructive sleep apnea. OSA severity was classified by the AHI derived from polysomnography (AHIPSG). The total number of participants was 44. The number of participants with AHIPSG≥5/h, AHIPSG≥15/h, and AHIPSG≥30/h was 36, 22, and 9, respectively. AHI, apnea–hypopnea index; AUC, area under the curve; OSA, obstructive sleep apnea.

The performance of specific AHI_PAT_ cutoffs (≥5/h, ≥15/h, and ≥30/h) in identifying all, moderate-to-severe, and severe OSA was first tested (Table 3). AHI_PAT_ ≥ 15/h identified moderate-to-severe OSA with a sensitivity of 77.3% (95%CI 54.2–91.3%) and a specificity of 100.0% (95% CI 81.5–100.0%). ROC curves were then constructed to evaluate sensitivity and specificity at different thresholds (Figure 3). The areas under the curves (AUCs) were 0.87 (95% CI 0.74–0.95), 0.92 (95% CI 0.80–0.98), and 0.87 (95% CI 0.73–0.95) for all, moderate-to-severe, and severe OSA, respectively.

**Table 3:** Sensitivity and specificity data for Watch-PAT in the diagnosis of obstructive sleep apnea among individuals with chronic insomnia.

Diagnostic performance	AHI_PAT_ ≥ 5/h for all OSA	AHI_PAT_ ≥ 15/h for moderate-to-severe OSA	AHI_PAT_ ≥ 30/h for severe OSA
Sensitivity (%)	81.8 (95% CI 66.8–91.3)	77.3 (95% CI 54.2–91.3)	33.3 (95% CI 9.0–69.1)
Specificity (%)	50.0 (95% CI 17.4–82.6)	100.0 (95% CI 81.5–100.0)	100.0 (95% CI 87.7–100.0)
Positive predictive power (%)	88.9 (95% CI 73.0–96.4)	100.0 (95% CI 77.1–100.0)	100.0 (95% CI 31.0–100.0)
Negative predictive power (%)	50.0 (95% CI 17.4–82.6)	81.5 (95% CI 61.3–93.0)	85.4 (95% CI 70.1–93.9)

Finally, the diagnostic agreement was also assessed using clinically oriented criteria. Agreement of AHI was reached in 36 (82%) subjects. In the remaining eight subjects, Watch-PAT uniformly underestimated AHI and overestimated TST in six subjects. After adjustment for TST, the AHI_PAT_ still complied with the underestimation criteria in five of these six subjects.

## Discussion

The current study evaluated the performance of a wrist-worn HSAT for diagnosing obstructive sleep apnea in patients with chronic insomnia. The study demonstrated that across a wide range of sleep duration, latency, and efficiency, the Watch-PAT-derived AHI achieved diagnostic agreement with PSG in over 80% of subjects and detected moderate-to-severe OSA with high specificity.

In clinical practice, HSAT is most frequently used for diagnostic purposes among patients with suspected moderate-to-severe OSA. In the present insomnia cohort, Watch-PAT achieved an AUC of 0.92 on the ROC curve, identifying moderate-to-severe OSA. This performance was similar to that observed in unselected sleep clinic and population-based cohorts (14, 21). An HSAT-specific agreement analysis was also performed to detect clinically significant discordances between Watch-PAT and PSG-derived AHI values. Such a clinically defined agreement was reached in 82% of participants, approaching the accuracy observed in a chronic obstructive pulmonary disease (COPD) cohort (23). Applying AHI_PAT_ ≥ 15/h as the threshold, the sensitivity and specificity of Watch-PAT for detecting moderate-to-severe OSA were 77.3 and 100.0%, respectively. This sensitivity indicates that approximately one in four moderate-to-severe OSA cases may be missed or categorized into a lower severity tier by Watch-PAT. This finding is further highlighted by the analysis of the eight clinically discordant cases, all of which were underestimated by the Watch-PAT device. Moreover, this underestimation persisted in the majority of cases after adjustment for sleep time. In another comparative study between Watch-PAT and PSG, the frequency of underestimation also far exceeded that of overestimation (18.2% vs. 3.0%) (23). Thus, this pattern may represent an intrinsic characteristic of the Watch-PAT method rather than merely reflecting selection bias.

In the present study, Watch-PAT overestimated TST and underestimated WASO in patients with insomnia. As is known, the assessment for OSA severity, either by conventional (e.g., AHI and oxygen desaturation index) or newly defined parameters (e.g., hypoxic burden and sleep breathing impairment index), relies profoundly on the accurate recording of sleep time (24). In previous validation studies, the Watch-PAT embedded actigraphy provided an unbiased estimation of TST, with agreement above 80% in epoch-by-epoch comparisons with PSG (12). In this study, Watch-PAT overscored sleep by an average of approximately 20 min (or 5% of TST). Such overscoring of sleep can be explained by the scenario that individuals lie awake with minimal wrist movement (25). Indeed, most studies conducted among individuals with insomnia have documented overestimation of sleep duration by actigraphy (26). Nevertheless, this discrepancy has decreased with the evolution of scoring algorithms (26, 27).

As an HSAT system without electroencephalography, Watch-PAT distinguishes REM from non-REM sleep based on vasoconstriction and resultant PAT signal changes (11). It is well understood that during REM sleep, respiratory physiology is entirely altered, and the propensity for upper airway collapse is significantly increased. The rules for keeping and adding respiratory events in the Watch-PAT manual scoring are more permissive during REM sleep due to the reduced PAT amplitude (21). As a result, REM staging in Watch-PAT directly affects the AHI measurement. In the present investigation, Watch-PAT overestimated REM duration by a considerable extent (mean bias 37.2 min, mean REM duration 59.3 min). The agreement between PSG- and Watch-PAT-derived REM duration was also poor (ICC = 0.25, 95% CI -0.09–0.54). These results are consistent with previous validation studies conducted in unselected clinical populations (21) and raise concerns regarding the use of Watch-PAT-derived sleep staging among individuals with insomnia. Furthermore, respiratory events predominantly occurring during REM sleep may represent a distinct subtype of OSA of clinical importance (28, 29). The current study indicates that the REM-specific AHIs measured by Watch-PAT show large discrepancies compared with PSG in individuals with insomnia and should be interpreted with caution in clinical practice.

With a deeper understanding of the interaction between insomnia and OSA, there is a growing trend toward integrating the assessment and management of both conditions. The experience reported here with Watch-PAT suggests a possible model in which objective sleep monitoring, OSA diagnosis, and treatment outcome measurement can be accomplished entirely using home-based instruments. This model may be implemented through a combination of HSAT and actigraphy. The incorporation of evolving wearables and nearables would provide more options for disease monitoring and intervention (30). In addition, combining device-derived sleep and respiratory parameters with targeted biological markers has been shown to enhance the ability to predict OSA severity and treatment response (31). The integration of minimally disruptive sleep-monitoring devices and the application of machine learning to device-derived data may represent a promising direction for the personalized management of COMISA (32).

Several potential limitations of this study need to be discussed. First, participants were recruited from visitors to a sleep breathing disorders clinic, whose chief complaints were primarily suggestive of OSA rather than insomnia. Consequently, the most severe cases of insomnia were likely underrepresented. Nonetheless, the study covered a wide range of sleep parameters, including TST, sleep latency, and sleep efficiency. Second, prevalent comorbid conditions associated with insomnia (e.g., restless legs syndrome and periodic limb movements in sleep) were not systematically screened for or excluded. The potential presence of these conditions in our cohort may have contributed to sleep fragmentation and insomnia symptoms, acting as unmeasured confounding variables. Third, due to the limited number of participants, only nine individuals with severe OSA were analyzed, and the maximum AHI was approximately 80/h. As the severity of OSA may affect the accuracy of sleep/wake assessment by Watch-PAT (12), extending the findings of the current study to more severe cases should be made with caution. Finally, the sleep/wake scoring was not analyzed in an epoch-by-epoch manner. However, as the objective of the current study was to assess the overall performance of Watch-PAT as a diagnostic tool, the precise accuracy of Watch-PAT in sleep/wake scoring was not the focus. Despite these limitations, the current study represents a preliminary attempt to validate ready-to-use HSATs in individuals with COMISA, an important yet underrecognized clinical scenario.

## Conclusion

In this preliminary study of a small population, Watch-PAT achieved an AUC of 0.92 on the ROC curve for identifying moderate-to-severe OSA in individuals with chronic insomnia. Nevertheless, the wrist-worn device appears to systematically underestimate the AHI while overestimating total sleep time and REM sleep duration. In the assessment of comorbid insomnia and sleep apnea, Watch-PAT-derived data should be interpreted with caution.

## Data Availability

The raw data supporting the conclusions of this article will be made available by the authors, without undue reservation.
